# Case of Cancer, Cured by Repeated Excisions and a Diet of Indian Corn, (Zea Mays, Linn.)

**Published:** 1832-04

**Authors:** Leonard Peirce

**Affiliations:** Sutton, Mass.


### CANCER.

*Case of Cancer, cured by repeated Excisions and a Diet of Indian Corn,* (*Zea mays, Linn.*) By LEONARD PEIRCE, M.D., of Sutton, Mass.*

THE pathology and treatment of cancer are still involved in much uncertainty. The investigation of its causes, with the best mode of treatment, has for a long period of time employed some of the ablest minds of which the profession can boast, but notwithstanding the numerous explanations which have been given of its causes, and the still more numerous methods of cure, embracing nearly every variety, from the most simple and cautious to the most complicated, the disease is still where it was centuries ago, in the first rank of the most formidable and terrifying of human maladies. Its bare name is associated with almost certain death: at the first intimation of its approach, life recoils with a wish that it had never been called into being; and, on its actual existence, fortitude itself vanishes, and leaves the mind a prey to the most gloomy anticipations. Whoever shall be so fortunate as to point out a method of treatment which will ordinarily prove successful, may rest satisfied that, without any other monument, his name will be remembered with gratitude in all enlightened countries, so long as cancer shall continue to afflict his fellow-creatures. However unsuccessful the efforts of surgeons may have been, it is still their duty never to relax their labours so long as a chance remains for improvement.

In offering a history of the following case to the profession, I shall not, perhaps, present any thing entirely new, yet I believe some portion of the treatment is different from any which has ever been recommended by writers on the subject, and, from the entire success which seems to have attended it, is entitled to a respectful consideration. But should I fail of presenting any thing new, and offer nothing but the details of an ordinary case, I shall readily be pardoned for the interest I may manifest in its relation, from the subject of it being my mother. Sufficient time has elapsed since the last operation, to inspire considerable confidence in the success of this course of treatment; and should it be

* American Journal of Med. Sciences.

adopted by others, it is hoped the result will be made public.

The disease was in the right breast. She first discovered a tumor about the size of a chesnut, in September 1825, at which time she was in her sixty-second year. On the following March she consulted me, and I recommended its removal, but, on the recommendation of Dr. SMITH, of this town, concluded first to try the effects of iodine. I commenced with fifteen drops of the tincture of iodine three times a day, in a little molasses and water, and gradually increased the dose eleven drops: at the same time the iodine ointment was applied to the whole breast two or three times a day. This treatment was continued for several weeks, and the tumor continued to grow, attended with lancinating pains in the breast and axilla; and it was thought by Drs. GREENE and HEYWOOD, of Worcester, and Dr. SMITH, of this town, that its removal should be delayed no longer. It was removed on the 29th of May, 1826, by Dr. John Greene, assisted by Drs. Heywood, Smith, and myself. The patient sat in a chair, with the arms carried back by an assistant, but otherwise remained at liberty during the whole operation, which she bore with remarkable fortitude. The head rested upon a pillow supported by myself. Two incisions were made in the direction of the axilla and sternum, including sufficient width of skin to make a level surface after the gland was removed; the whole of which was taken away, together with a part of the fascia covering the pectoral muscle. The lips of the wound were drawn together with adhesive straps, and dressed with simple cerate. This wound healed without any difficulty, and in the usual time.

After recovery from this operation, her health remained as good as usual, until the winter of 1826 and 7, when another tumor appeared just beneath the cicatrix, attended with pains darting into the axilla, as in the first tumor: similar pains were now felt in the neck of the uterus. No medical treatment was now adopted, and the only reliance was upon the scalpel. It was removed on the 5th of April, 1827, by Dr. Smith, assisted by myself. A simple incision was made, the tumor not being more than an inch in diameter. The wound was dressed with adhesive straps and cerate as before, and the dressings renewed every day. It shewed but little disposition to heal, and luxuriant granulations shot up from every part of it, and a very little purulent matter was discharged.

Within a few weeks from the removal of the last tumor, two others appeared in the neighbourhood of the wound, increased rapidly, soon ulcerated, united with the granulations, and formed a hemispherical tumor, which was slightly elevated above the surrounding skin, and seemed to rest on a partial peduncle. The centre of the tumor appeared to increase faster than the borders, which were thus made tense, and turned in towards the peduncle. The whole surface of the tumor had an exceedingly jagged and angry appearance: it would bleed on the slightest injury, and often without any hurt whatever. The discharge was not now at all purulent, but watery, giving to the dressings a very slight tinge of yellow, and rendering them a very little stiff when dry. Whenever this discharge came in contact with the sound skin, it produced an intense smarting, and, unless immediately wiped off, would excoriate. If the dressings were suffered to remain after they became wet with the discharges, the pain was increased to an insufferable degree, and she was obliged to change them every ten or fifteen minutes during the whole time. The pain in the tumor and axilla had now become exceedingly severe, and almost uninterrupted: it was severe, but less constant, in the neck of the uterus. Death now seemed to be the inevitable consequence of the disease. All the appearances were more unfavorable than heretofore, and she declined having it removed again, from a belief that it would be attended with no better result than before, and that it would only add to her sufferings, which must soon terminate her life.

I now wrote to Professor Smith, of Yale College, whose pupil I had been, giving him a history of the case, with the treatment which had been pursued, requesting his opinion of the case: he replied as follows.

"*New Haven; August* 11, 1827.

"Dear Sir: Yours of the 1st instant has been received. Respecting your mother's case, I would advise to remove all the tumor at once. I once removed a cancerous breast which weighed seven and a half pounds. Several tumors arose afterwards in the neighbourhood of the cicatrix, which were removed, and the woman is now living and well.

"After removing the tumors, I would put her upon a vegetable diet, and not allow her to taste animal food for a year at least. I should like to have her live some time on green corn and water. She may boil it or roast it as she likes it best, or she may cut it off the cob and boil it in water and season it with salt, and eat it in that way. If you cut it from the cob, do it as follows: Take the ear fresh picked, and having a pan of cold water, put the end of the ear on the bottom of the pan, and with a knife cut off the corn, and let it fall into the water. Scrape the cob with the knife and plunge it in the water, and wash out all the milk that adheres to it. I have found that a diet of Indian corn is very favorable to cancerous patients.

"I would give the following medicine: ℞. Red Oxide of Iron, ℥iss.; Ext. Conium, ℥ss.; Simp. Syrup, q. s. Mix and make twelve pills to a drachm. Dose to commence with three pills morning, noon, and night, and increase the dose to as large a quantity as she can bear on account of the conium.

"I am, with sentiments of esteem, your friend, &c.

"Nathan Smith."

"Leonard Peirce, m.d."

She would not yet consent to the removal of the tumor, but commenced immediately with the diet, which she most rigidly adhered to. The corn was prepared by cutting it from the cob, and boiling it as directed. She used the oxide of iron and conium in as large quantities as she could bear.

Drs. Green and Smith were now called, who united with the opinion of Professor Smith in recommending its removal, to which she finally consented, more however in deference to the opinions of her advisers, than from a belief that it would arrest the disease. She now had an attack of the dysentery, from which she did not recover for two or three weeks. During the acute stage of this disease the discharge from the cancer considerably abated, but was not otherwise affected. On the 13th of September she had so far recovered as to be able to submit to the operation. At this time the tumor was larger than the breast, whose place it occupied, and it had for more than two months bled from one to three ounces daily. The skin around the base or peduncle, which was now nearly four inches in diameter, was so diseased as to require its removal to the distance of nearly an inch on each side of it. Her health was very feeble from previous suffering, and much doubt was entertained of her surviving the operation. She was seated in a chair as at first, and supported, but not confined by assistants as before. Dr. Greene operated, assisted by Drs. Heywood, Smith, and myself. Two incisions were made, meeting at the sternum and axilla. The tumor was cautiously and with much dexterity dissected from the pectoral muscle, which appeared to be perfectly sound and healthy. It was thought that, could the lips of the wound be brought together, it would be more likely to heal than it would if a large surface was left uncovered by skin. By considerable effort the lips were brought together, and confined by the interrupted suture and adhesive straps. This put the skin so violently upon the stretch, that her head, neck, and shoulders were drawn obliquely forwards and downwards, which occasioned for a time severe pain. The operation was performed about three o'clock in the afternoon, and notwithstanding the force used in the dressing, she passed the succeeding night more comfortably than she had any night for six weeks before.

Nothing unfavorable took place to interrupt the healing of this wound, except some slight granulations shooting upon its edges, which were reduced by the daily application of nitrate of silver. It soon entirely healed, and formed a round cicatrix, and the pains in the axilla and uterus had entirely subsided. She had up to this time most rigidly adhered to her prescribed diet, eating nothing but boiled corn seasoned with salt, and drinking nothing but simple and cool drinks. The pills were omitted after the operation. She was fully convinced that she was deriving essential benefit from her diet, and, notwithstanding the severe self-denial it imposed upon her, most cheerfully submitted and clung to it as her only hope.

When corn was in a fit state to boil, she had a large quantity of it gathered, boiled, dried in the sun, and laid up for future use. Prepared in this way, it would not receive injury by keeping, and retained the flavour of corn fresh picked. On this she subsisted till the next season for green corn returned, when she supplied herself again from the field, and continued its use during the season for it. She then began the use of boiled ripe corn, sometimes boiling it as it came from the cob; at others, having it cracked in the mill, and made into homony, which she ate with molasses. She frequently ate, instead of the corn, bread made of Indian meal, mixed with water, and baked by the fire.

She now attempted the use of animal food, by barely tasting it, but was obliged to abandon it at once, as it produced pains in the axilla, similar to those she had felt in the cancer; it likewise made her mouth and throat sore. The attempt was several times renewed at intervals of several months, but always with similar results. Roasted potatoes agree with her well. She occasionally eats fish sparingly. She continues her diet to this time, and most studiously avoids all kinds of animal food, all kinds of distilled or fermented liquors, spices of every kind, and all hot and heating drinks. Exercise and mental emotions, for a considerable time after the last operation, produced effects similar to those of animal food, and she most sedulously avoided them. She has never deprived herself the use of tea, but always takes it cool, and in limited quantities. From the time she began to diet, the alvine discharges were thin and watery, and continued so long as she confined herself to green corn; they were likewise, for a considerable time, hot and excoriating. Within a few months from the last operation, she was able to sit erect, and had a tolerably free use of her right arm, and its motions are now entirely free. During the time she confined herself to green corn, her strength was so feeble that she was unable to perform any manual labour, but took the general superintendence of her household affairs. After she began the use of ripe corn, and eat bread and other vegetables, her strength increased so much that she was able to do some light work. At present, her health is as good as before she was afflicted with cancer, and she has become so accustomed to her diet, that she enjoys it as well as she would a more generous one.

